# Electronic School Absenteeism Monitoring and Influenza Surveillance, Hong Kong

**DOI:** 10.3201/eid1805.111796

**Published:** 2012-05

**Authors:** Calvin K.Y. Cheng, Benjamin J. Cowling, Eric H.Y. Lau, Lai Ming Ho, Gabriel M. Leung, Dennis K.M. Ip

**Affiliations:** The University of Hong Kong, Hong Kong Special Administrative Region, People’s Republic of China

**Keywords:** human influenza, school absenteeism, electronic surveillance, influenza-like illness, public health, influenza, viruses, Hong Kong

**To the Editor:** Potentially useful public health interventions, such as school closure, need to be introduced in a timely manner during the evolution of an ongoing epidemic to substantially affect community transmission (*1**,**2*). In most traditional surveillance systems that include health care use data, however, considerable delays occur between data collection and feedback, which leads to suboptimal and untimely information for guiding evidence-based public health decisions. Newer syndromic surveillance approaches have been attempted to improve timeliness by targeting earlier events in the health-seeking pathway and by promoting real-time collection and processing of surveillance data by using modern information technology (*3**,**4*). Building on an existing platform of an electronic school management system, we developed an automated school absenteeism surveillance system for influenza-like illness (ILI) in Hong Kong and evaluated its performance using data collected from March 2008 through June 2011. The Institutional Review Board of the University of Hong Kong/Hospital Authority, Hong Kong West Cluster, approved the study.

We collaborated with a commercial vendor that develops and provides online learning platforms and management systems for educational institutions, including 337 primary and secondary schools in Hong Kong, attended by children 6–18 years of age. Invitations to participate in the new absenteeism system were sent to all schools subscribing to the electronic school management system, and 62 schools throughout Hong Kong were recruited in phases during the study period. We began with 18 schools (17,255 students) from February through June 2008, then expanded to 45 schools (37,087 students) in 2008–09, to 50 schools (41,765 students) in 2009–10, and to 62 schools (49,425 students) in 2010–11.

The absenteeism system worked as follows. A student identification smart card was issued to each student in all participating schools, and students were required to swipe their cards over a sensor at the school entrance as an electronic record of attendance, which replaced a traditional paper-based roll call. Reasons for absence, including ILI, were asked in telephone calls in a subset (37%) of schools, and answers were manually entered into the system by teachers. Daily aggregated data, including the total number of students and number of absentees in each grade (stratified by reason for absence), were compiled each afternoon. Individual children were not identifiable. Data cleaning, aggregation, and analysis and generation of reports were automated with R version 2.12.1 (R Foundation for Statistical Computing, Vienna, Austria). Weekly overall absenteeism rates were calculated as the total number of absentees divided by the total number of students at all schools. Regular weekly and ad hoc reports of absenteeism patterns, with an interpretation of the overall influenza disease activity in the community, were distributed to all participating schools through the same school management system and disseminated to the general public through an existing influenza surveillance dashboard (*5*).

The school absenteeism rates and reference data from 2 existing traditional surveillance systems in Hong Kong during March 2008–June 2011 covered a total of 7 influenza seasons (Figure). Data from both systems were from all age groups in the entire territory. The laboratory virus isolation rate as a reference standard highlighted the typical seasonality of influenza in Hong Kong, which peaked around February during winter and around August during summer, in each year (Figure, panel D) (*6*). Clear and sharp peaks were detectable from both the overall and ILI-specific data during most of these influenza seasons (Figure, panels A, B), which generally occurred 1–3 weeks ahead of the peaks in the laboratory data (range 1 to 5 and −1 to 4 weeks; median 3 and 0.5 weeks for overall and ILI data, respectively). The data generally showed much sharper peaks than the outpatient sentinel data, possibly related to better coverage of disease activity in the community, in contrast to the sentinel data, which captured only episodes leading to visits to outpatient clinics (*7*). Limitations of our school absenteeism data included relatively lower perceived sensitivity of the ILI-specific absenteeism rate (because data are provided only by 37% of participating schools) and the presence of data gaps during school holidays or school closure as a result of public health measures to mitigate seasonal influenza in March 2008 (*1*) and pandemic influenza in June 2009 (*2*).

**Figure:**
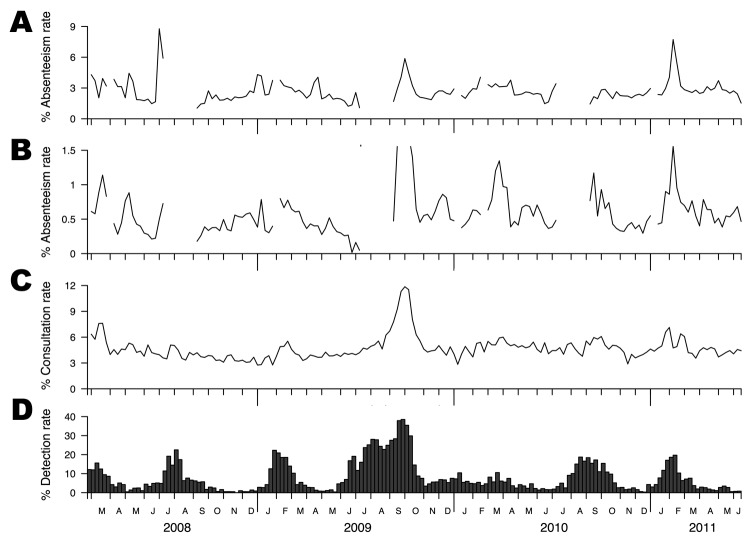
Influenza surveillance data, Hong Kong, February 23, 2008–June 18, 2011. A) Weekly overall school absenteeism rate. B) Weekly influenza-like illness (ILI)–specific school absenteeism rate. C) Weekly ILI (defined as fever plus cough or sore throat) consultation rates in sentinel networks of outpatient clinics in the private sector. D) Proportion of influenza A and B virus isolations (by date of collection) among all specimens submitted to the reference laboratory for Hong Kong Island at Queen Mary Hospital.

This study demonstrated the feasibility and potential benefit of using automatically captured school absenteeism data as a complementary data stream for influenza surveillance. Real-time monitoring of school absenteeism, an early event in the health care–seeking pathway, can improve situational awareness and help inform appropriate public health decisions and interventions in a more timely and evidence-based manner. Electronically capturing data from preexisting smart card systems is an attractive and cost-effective option that does not require substantial additional resources, systems, or labor in contrast to some other approaches (*8**–**10*). The increasing popularity of smart card technology in various situations might also provide potential opportunities for innovative surveillance systems.
